# Cytomegalovirus viremia in an 11-year-old child with sickle cell disease manifested only with fever: a case report

**DOI:** 10.1097/MS9.0000000000000877

**Published:** 2023-06-08

**Authors:** Mahfoud EID, Abd Ghanem, Elias Saloum

**Affiliations:** a Department of Pediatrics; b Department of Pediatrics; c Faculty of Human Medicine, Tishreen University, Latakia, Syria

**Keywords:** cytomegalovirus, fever of unknown origin, sickle cell disease

## Abstract

Cytomegalovirus (CMV) is a widespread virus, and it affects people of all ages. Infection with this virus causes severe life-threatening disease in immunocompromised patients and newborns. While CMV infection is asymptomatic or present as a mild illness in most cases of immunocompetent patient, it may cause severe diseases in 10% of cases.

**Case presentation:**

Here, the authors describe the case of an 11-year-old male presented with an ischemic stroke due to sickle cell disease; who, during hospitalization, developed a prolonged fever. After excluding bacterial infections, infiltrating diseases, rheumatologic diseases, malignancies, and other possible causes, he was diagnosed with CMV infection, which not checked initially, because most cases are asymptomatic.

**Conclusion:**

This case highlights the need to consider CMV infection in the differential diagnosis of every case of fever of unknown origin, regardless of the patient’s immune status.

## Introduction

Highlights

- Human Cytomegalovirus (CMV) is typically asymptomatic or may cause a self-limited disease in healthy children.
- In rare cases, human CMV may cause life-threatening infection in immunocompetent children.
- Fever is a common presenting complaint in children.
- Viral infections may be more virulent in individuals with sickle cell disease.
- We should keep CMV infection in the differential diagnosis of fever of unknown origin.

Human Cytomegalovirus (CMV) is a worldwide virus that commonly infects people of all ages throughout the world^1^. Human CMV is a member of the Herpes-virus family, which have a genome of double-stranded linear DNA, and biological properties of latency and reactivation, which cause recurrent infections in the host. They also have a viral envelope and virus capsid icosahedral symmetry^2^.

Infection with CMV occurs commonly, and children infected with CMV may acquire the virus during the toddler or preschool years, especially if they are in contact with other children in a group setting^1^. The clinical manifestations of CMV infection in infants and children depend upon the age and immune status of the patient, ranging from an asymptomatic form and a mild disease in immunocompetent patients to a severe and life-threatening form in the immunocompromised and newborns^1^.

Fever of unknown origin (FUO) refers to children with fever greater than 38.3°C (101°F) of at least 8 days’ duration with no apparent diagnosis after initial evaluation that includes a detailed history, thorough physical examination, and initial laboratory assessment^3^. FUO is usually caused by common disorders, often with an unusual presentation^4^. The most common causes are bacterial and viral infections^5^. Although most viruses cause short term self-limited illness. However, Epstein-Barr virus, CMV, and hepatitis viruses, can cause FUO.

The main objective of this article is to highlight the importance of placing CMV infection within the differential diagnosis of FUO, regardless of being asymptomatic in most cases and regardless of the patient’s immune status.

This case report has been reported in line with the Surgical CAse REport (SCARE) criteria 2020^6^.

## Case presentation

An 11-year-old male presented in our emergency room with lethargy, vomiting, weakness, left peripheral facial nerve paralysis, and left Hemiparesis signs. He was the first child of nonconsanguineous parents, born at term after a normal pregnancy. His past medical history revealed sickle cell disease (SCD), diagnosed at the age of 2 years by hemoglobin (HgB) electrophoresis, and he was on folic acid only. He had received all of his immunizations along with the pneumococcal and meningococcal vaccine. He had frequent hospitalization in the context of acute painful episodes, as well as blood transfusions. He underwent a tonsillectomy at the age of nine years. His family history showed that both parents were sickle cell carrier.

At admission to the PICU, he was pale and in reduced general condition. On physical examination, he had a normal heart rate with 100 beats/min and 2/6 murmur. Detailed neurological examination showed left-sided paralysis with increased deep tendon reflexes and a positive Babinski sign, rising concern of a cerebral stroke. There was no adenopathy nor hepatosplenomegaly. The rest of his physical examination was unremarkable.

His initial vital signs were: respiratory rate: 25 breaths/min, Spo_2_: 93% through a nasal canal 1 l/minute, blood pressure: 100/60 mmHg, and temperature: 37°C. His weight was 25 kg.

His initial laboratory workup showed elevated white blood cells (WBCs) and platelets count, decreased HgB, and elevated lactate dehydrogenase [Table 1].

**Table 1:** The initial workup of the patient upon admission

Test	Result	Normal values	Test	Result	Normal values
WBC	23×10^3^ **↑**	4.5-11× 10^3^/μl	CRP	2	0–6 mg/dl
Neutrophil	82%**↑**	50–70%	Creatinine	0,8	Less than 1.4 mg/dl
Lymphocytes	12%**↓**	20–40%	BUN	20	7–35 mg/dl
Hemoglobin	8,5**↓**	More than 11 g/dl	ALT	19	7–55 U/L
Platelets	646×10^3^ **↑**	150–450×10^3^/μl	LDH	1283**↑**	60–170 units/L

ALT, Alanine transaminase; BUN, Blood urea nitrogen; CRP, C-reactive protein; LDH, Lactate dehydrogenase; WBC, Wight blood cell.

A brain computed tomography (CT) was performed, and was within normal. A chest radiograph was also done, and it was normal.

He underwent a partial blood exchange (20 ml/kg), then we initiated the treatment with Hydroxyurea (20 mg/kg/dose once daily).

By day 2, a brain CT was repeated, which showed a hypodense area in the right temporoparietal region, confirming ischemic stroke.

By day 3, the patient developed a fever of 40°C, at a rate of 3 peaks/day. Biochemical workups were normal except for elevated WBCs, with decreased HgB. A chest radiograph was performed, which showed bilateral infiltrates. He was commenced on vancomycin, ceftazidime, and clarithromycin.

**Figure 1:**
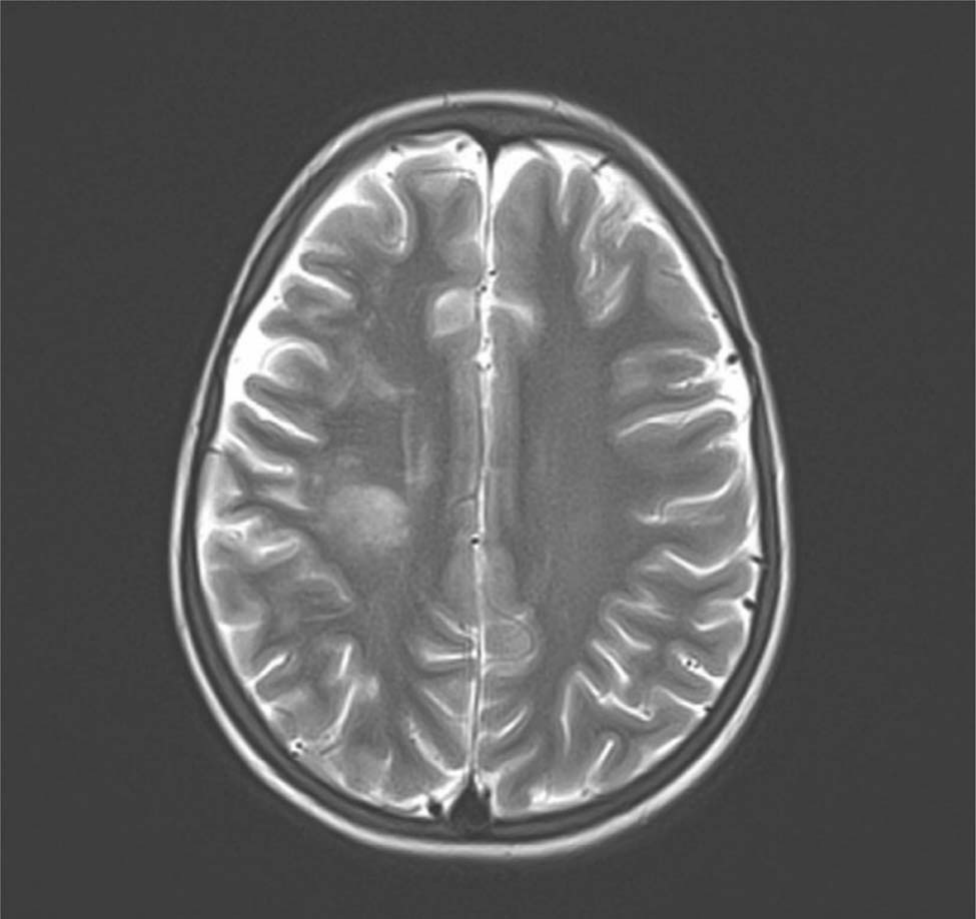
T2-weighted brain MRI showing right sub-acute temporoparietal infarction.

By day 10, the patient was weaned off oxygen with slight improvement in upper and lower left extremity movement on physiotherapy, but the fever persisted with nonobvious cause which is consistent with FUO. A complete blood count, C-reactive protein (CRP), blood culture, serologic test for typhoid fever, and brucellosis were done [Table 2], as well as MRI in order to investigate a potential cerebral abscess, which showed a right sub-acute temporoparietal infarction [Fig. 1].

**Table 2:** Tests’ results of the patients in day 10 of hospitalization

Test	Result	Normal values	Test	Result	Normal values
WBC	54×10^3^ **↑**	4.5-11× 10^3^/μl	ALT	51	7–55 U/L
Neutrophil	66%	50–70%	AST	37	10–40 U/L
Lymphocytes	25%	20–40%	Widal	Neg	Neg
Hemoglobin	6,5**↓**	More than 11 g/dl	Wright	Neg	Neg
Platelets	470×10^3^ **↑**	150–450×10^3^/μl	Blood culture	Neg	Neg
CRP	212**↑**		0–6 mg/dl		

AST, Aspartate transaminase; ALT, Alanine transaminase; CRP, C-reactive protein; WBC, Wight blood cell.

Based on the results of Table 2, which showed a markedly elevated WBCs, decreased HgB, and elevated CRP; a simple blood transfusion was performed and the patient was commenced on a broad-spectrum antibiotic. An episode of hemolysis occurred during the transfusion; therefore, it was immediately discontinued. A direct-coombs (IgG) test was negative, but the indirect-coombs test was positive, which is consistent with alloimmune hemolytic anemia. The hemolysis was mild and self-limited, so we did not need using immunosuppressive therapy. Conversely, we had difficulty accessing to compatible blood for subsequent transfusions, which prompted us to use Vitamin B9 and Vitamin B12.

By day 20, the fever remained elevated (40°C), with 2 peaks/24 h. A complete blood count, CRP, erythrocyte sedimentation rate, procalcitonin, serologic test for typhoid fever and brucellosis was performed [Table 3], as well abdominal ultrasound which excluded any visceral abscess, and an echocardiogram, which rule out infective endocarditis, but it showed an important pericardial infusion. A whole-body CT scan was performed, in which nodular hyperplasia was observed in the neck, chest, abdomen, and pelvis.

**Table 3:** Tests’ results of the patients in day 20 of hospitalization

Test	Result	Normal values	Test	Result	Normal values
WBC	19×10^3^ **↑**	4.5-11× 10^3^/μl	CRP	50**↑**	0–6 mg/dl
Neutrophil	68%	50–70%	ESR	20**↑**	Less than 10 mm/hr
Lymphocytes	27%	20–40%	Procalcitonin	0,17	Less than 0.5 ng/ml
Hemoglobin	7,6**↓**	More than 11 g/dl	Wright	Neg	Neg
Platelets	625×10^3^ **↑**	150–450×10^3^/μl	Widal	Neg	Neg

CRP, C-reactive protein; ESR, Erythrocyte sedimentation rate; WBC, Wight blood cell.

Following results presented in Table 3, which showed elevated WBCs, platelet count, CRP, and erythrocyte sedimentation rate, and decreased HgB; he underwent a bone marrow needle aspiration, to rule out malignancy or any other infiltrate disease, it showed a reactive hyperplastic bone marrow without abnormal cells. The antibiotics were stopped for 72 h to detect pharmacotherapy fever. Despite this, the fever persisted, and viral and rheumatologic causes were raised, as well as inflammatory bowel disease.

Coronavirus disease 2019 PCR, anti Hbs-Ag, HCV PCR, CMV IgM, fecal calprotectin, antinuclear antibodies, antidouble-stranded DNA antibodies, antineutrophil cytoplasmic antibodies test, were ordered [Table 4].

**Table 4:** Tests’ results of the patients after 23 days of hospitalization

Test	Result	Test	Result
CMV IgM	2,34**↑**	Anti HCV IgM	Neg
CMV IgG	3 U/ml (Normal up to 7)	Fecal calprotectin	Neg
CMV PCR	113 000**↑**	ANA	Neg
Covid-19 PCR	Neg	Anti-ds-DNA	Neg
Anti Hbs-Ag	Neg	ANCA	Neg

ANA, Antinuclear antibodies; ANCA, Antineutrophil Cytoplasmic Antibodies; Anti-ds-DNA, antidouble-stranded DNA antibodies; CMV, Cytomegalovirus; HCV, Hepatitis C virus.

According to the findings presented in Table 4, which showed a positive CMV IgM (2.3 Ru/ml, normal value: up to 0,5 Ru/ml), and the diagnosis of CMV infection was made after confirming the result with a positive CMV PCR (113 000 copies/ml, undetectable: up to 100 copies/ml) [Table 4]. The patient was commenced on Ganciclovir (5 mg/kg every 12 h for 14 days), and the fever disappeared after 4 days of treatment.

By day 37, after the improvement in general condition and mobility of the patient’s left extremities, he was discharged with a recommendation to continue physiotherapy and hydroxyurea.

## Discussion

Our case described an 11-year-old child, who diagnosed with CMV infection, an infection which is most often asymptomatic in healthy children and adolescents, and only ~10% of cases produce symptoms.

CMV can cause a mononucleosis–like syndrome, with being fever, pharyngitis, fatigue, adenopathy (especially cervical adenopathy), and hepatitis, the most common manifestations; along with biochemical abnormalities that include lymphocytosis or lymphopenia with thrombocytopenia and elevated transaminases^7^. In our case, the fever was the unique manifestation of this infection.

Acquired CMV infection in healthy individuals may present with unusual manifestations and complications^1,^ as described by many case reports in literature, including, pneumonitis (Yinghu Chen *et al*.)^8,^ hemolytic anemia (Hyun Jung Hong, *et al*.)^9,^ myocarditis, and Meningoencephalitis (E Bäck *et al*.)^10^.

Our patient had alloimmune hemolytic anemia demonstrated by a positive indirect-coombs test. Individuals with SCD have a high risk of alloimmunization and delayed hemolytic reactions, which occurs in ~30% of patients who are transfused at least intermittently^11^. Patients with severe hemolysis may respond to immunosuppressive therapy such as intravenous immune globulin, and glucocorticoids; whereas mild cases do not need as in our case.

Infection with CMV occurs commonly, and the majority of children in developing country are infected by three years of age^1^. Several studies have attempted to identify risk factors for CMV reactivation in critically ill patients. Frantzeskaki FG *et al*.^12^ suggest that the frequency of red blood cell transfused during ICU stay and the degree of inflammation are an independent risk factor for reactivation. Direct transmission of the virus via blood products may be the underlying cause of reactivation of the infection, although transfusion-related Immunomodulatory effects cannot be excluded.

Our patient had an ischemic stroke, which we believe played a role in its CMV viremia, since many ICU admission diagnoses, such as sepsis, major surgery, trauma, and burns, may provoke CMV viremia^13^.

We would like to highlight on the role of hydroxyurea therapy in reducing the complications of SCD, through its role in increasing the level of fetal hemoglobin, which reduces sickle hemoglobin polymerization, sickling, and vaso-occlusion^14^.

We assure that, although CMV infection occurs primarily during childhood and is usually asymptomatic, but it still can cause severe diseases, including pneumonitis, potentially life-threatening hemolytic anemia, encephalitis, hepatitis, and colitis.

## Conclusion

Although CMV infection cases are often asymptomatic or may present as a mild non-specific illness, but we suggest considering it as a differential in every case of prolonged fever for which other causes have been ruled out, in order to establish an early diagnosis, reduce the incidence of complications, and shorten the recovery period.

## Ethical approval

This case report did not require review by the Ethics Committee Tishreen university hospital, Latakia, Syria.

## Consent

Written informed consent was obtained from the patient's parents/legal guardian for publication and any accompanying images. A copy of the written consent is available for review by the Editor-in-Chief of this journal on request.

## Sources of funding

This research did not receive any specific grant from funding agencies in the public, commercial, or not-for-profit sectors.

## Author contribution

M.E.: contributed in study concept and design, data collection and interpretation, and performing an extensive literature review; A.G.: contributed in data interpretation, and as a mentor and reviewer for this case report; E.S.: contributed in writing the paper. We would also like to thank neurosurgeon Ali Mhammad for his contribution in the interpretation of the Neuroimaging findings.

## Conflicts of interest disclosure

The authors declare that they have no financial conflict of interest with regard to the content of this report.

## Research registration unique identification number (UIN)

1. Name of the registry: Not applicable.
2. Unique Identifying number or registration ID: Not applicable.
3. Hyperlink to your specific registration (must be publicly accessible and will be checked): Not applicable.

## Guarantor

Mahfoud Eid.

## Provenance and peer review

Not commissioned, externally peer reviewed.
